# Oxidative stress evaluation of skeletal muscle in ischemia–reperfusion injury using enhanced magnetic resonance imaging

**DOI:** 10.1038/s41598-020-67336-4

**Published:** 2020-07-02

**Authors:** Yoshinori Kuroda, Hitoshi Togashi, Tetsuro Uchida, Kazuyuki Haga, Atsushi Yamashita, Mitsuaki Sadahiro

**Affiliations:** 1 Division of Cardiovascular Surgery, Department of Surgery II, Faculty of Medicine, Yamagata University, 2-2-2 Iidanishi, Yamagata, 990-9585 Japan; 2 Health Administration Center, Yamagata University, Yamagata, Japan; 3 Radiation Department, Yamagata University Hospital, Yamagata, Japan

**Keywords:** Valvular disease, Peripheral vascular disease

## Abstract

Acute extremity arterial occlusion requires prompt revascularization. Delayed revascularization induces ischemia–reperfusion injury in the skeletal muscle. Organ injury-induced oxidative stress is widely reported, and oxidative stress is heavily involved in ischemia–reperfusion injury. This study aimed to evaluate oxidative stress in ischemia–reperfusion rat models using 3-carbamoyl PROXYL enhanced magnetic resonance imaging (3-CP enhanced MRI). Ischemia–reperfusion injury was induced through clamping the right femoral artery in rats, with a 4-h ischemia time in all experiments. 3-CP enhanced MRI was performed to evaluate oxidative stress, and the rats were divided into 3 reperfusion time groups: 0.5, 2, and 24 h. Signal intensity was evaluated using 3-CP enhanced MRI and compared in the ischemia–reperfusion and intact limbs in the same rat. Furthermore, the effect of edaravone (radical scavenger) was evaluated in the 4-h ischemia—24-h reperfusion injury rat model. The signal intensity of the ischemia–reperfusion limb was significantly stronger than that of the intact limb, suggesting that oxidative stress was induced in the ischemia–reperfusion muscle. Edaravone administration reduced the oxidative stress in the ischemia–reperfusion limb. The signal intensity of the ischemia–reperfusion limb was stronger than that of the intact limb, presumably reflecting the oxidative stress in the former. 3-CP MRI examination shows promise for effective assessment of oxidative stress and may facilitate early diagnosis of ischemia–reperfusion injury.

## Introduction

Reperfusion of ischemic organs is essential in organ survival and functional recovery. However, reperfusion often leads to an inflammatory response, exacerbating organ damage. This phenomenon is termed ischemia–reperfusion injury^1^. Ischemia–reperfusion injury occurs in various organs. In ischemia–reperfusion, various mechanisms such as overproduction of ROS (reactive oxygen species), Ca^2+^ overload, pH alteration, and release of inflammatory cytokines occur^2^. In particular, overproduction of ROS causes oxidative stress and impairs membrane lipids, proteins, and DNA^1^. ROS also activates the transcription factor, NF-kB, which exacerbates inflammation^1^. Skeletal muscle has high metabolic activity and is therefore susceptible to ischemia–reperfusion injury. Acute lower extremity arterial occlusive disease is an emergency disease that can cause severe leg necrosis if the ischemia is not resolved quickly^3^. If ischemia resolution is delayed beyond optimal treatment time, ischemia–reperfusion injury occurs even if arterial blood circulation reconstruction of the lower limb is performed. Further, myonephropathic metabolic syndrome can develop, which is potentially fatal^3^. The involvement of ROS in ischemia–reperfusion injury in skeletal muscle has been clarified by (1) the inhibitory effect of damage by radical scavengers and enzyme inhibitors, (2) the evaluation by measuring products of ROS, and (3) the evaluation of in vitro and ex vivo ROS production using fluorescence^3^. Regarding management of skeletal muscle damage and subsequent multiple organ failure by ischemia–reperfusion of the lower extremities, there is an urgent need to establish a method for real-time in vivo detection of ROS and toxic molecules.

Electronic spin resonance (ESR) is the gold standard for detecting ROS. Detection of ROS produced by tissues during ischemia–reperfusion has been performed. However, it is difficult to obtain precise anatomical information using the L-band ESR imaging system. So far, the mouse represents the maximum animal size of the loop-cap resonator and the penetration depth of microwaves. Magnetic resonance imaging (MRI) is a useful diagnostic tool with excellent spatial resolution. It also provides detailed anatomical information on the whole body in a single examination. 3-carbamoyl-PROXYL (3-CP) is known to be T1-sensitive and has been used as a clinical contrast agent for MRI^4^. We have previously reported that MRI in combination with 3-CP is useful to evaluate the redox status in various organs^5^. The increased signal intensity (SI) of 3-CP in vivo is considered to represent an oxidative stress state^6^. Therefore, in the present study, we evaluated local oxidative stress in a model of ischemia–reperfusion injury in rat hind limb skeletal muscle using MRI in combination with intravenous administration of 3-CP. To the best of our knowledge, analysis of oxidative stress using MRI for ischemia–reperfusion injury has not been reported. Moreover, there are no clinical trials on the subject. We created a rat model of ischemia–reperfusion, analyzed MRI images after intravenous administration of 3-CP, and evaluated the usefulness of in vivo detection of oxidative stress. The purpose of the present study was to clarify whether a novel measurement method, i.e., MRI in combination with 3-CP, could detect oxidative stress in skeletal muscle in a real time manner after ischemia–reperfusion before the onset of skeletal muscle injury. Furthermore, we evaluated the effect of edaravone as a radical scavenger for oxidative stress occurring on ischemia–reperfusion injury muscle using 3-CP enhanced MRI.

## Methods

### Animals

All the care, maintenance, and experiments with animals were performed in accordance with the institutional guidelines and approved by the animal research Ethical Committee at Yamagata University (Yamagata, Japan).

Forty-four male Wistar rats aged 9 weeks (Japan SLC Inc., Shizuoka, Japan) were used in the study; their body weight was 210–250 g. All the animals were kept in stainless steel cages with standard pellet diet and tap water ad libitum and were maintained in a 12-h light–dark cycle. According to the ARRIVE guidelines, we randomly grouped the rats^7^.

### Ischemia–reperfusion injury model

All procedures were performed after subcutaneous administration of a 0.15 mg/kg medetomidine, 2 mg/kg midazolam, and 2.5 mg/kg butorphanol anesthesia cocktail. Following induction of anesthesia, the animal was placed in a supine position and the lower abdomen and right groin were shaved. A right groin incision was made and the right femoral artery was dissected. Ischemia was induced by clamping the right femoral artery with an atraumatic microvascular clamp. The branch arteries of the femoral artery were ligated as much as possible.

The ischemia time was set for 4 h in all experiments. The rats were divided into 4 groups (n = 4 each group): a no reperfusion group (A), a 0.5-h reperfusion group (B), a 2-h reperfusion group (C), and a 24-h reperfusion group (D). The animals were euthanized after reperfusion and samples of blood and skeletal muscle (both gastrocnemius muscles) were taken. The degree of organ injury was evaluated by measuring the plasma creatine kinase (CK) level and by histopathological analysis.

### Plasma creatine kinase level

The plasma CK level was determined to evaluate the degree of ischemia–reperfusion injury. Blood samples were taken from the heart before and after ischemia–reperfusion. The blood samples were centrifuged (3,000 rpm for 15 min at 4 °C) to obtain plasma. The plasma CK level was determined using a chemistry analyzer (Fujifilm Corporation, Tokyo, Japan) and compared before and after ischemia–reperfusion.

### Histopathological evaluation

Muscle biopsies were taken from both gastrocnemius muscles after ischemia–reperfusion. Tissues were placed in 10% formaldehyde and embedded in paraffin. The specimens were cut transversely at 5 μm on a microtome. Tissue sections were stained with hematoxylin and eosin and examined under light microscopy to compare the ischemia–reperfusion muscles with intact muscles.

The absolute injury score for each muscle was determined by a method similar to that of McCormack et al.^8^. Five visual fields (× 400) were randomly chosen on each slide and evaluated by two pathologists in a blind and independent manner. Myocytes were scored as uninjured or injured based on individual morphology. The muscle injury score was expressed as a percentage, obtained by dividing the number of injured myocytes by the total number of myocytes on all slides.

### MRI settings

The MRI examination was performed using Achieva 3.0-T Quasar Dual (Philips, Amsterdam, The Netherlands). All images were acquired with the following parameters: T1-weighted incoherent gradient-echo sequence, repetition time = 3.6 ms, echo time = 1.76 ms, flip angle = 10 degrees, field of view = 320 mm × 192 mm, number of averages = 1, scan time = 25.4 s, matrix = 480 × 480, slice thickness = 1.2 mm and number of slices = 140. We selected the coronal slices with a 0.67 × 0.67 × 1.2 mm^3^ nominal voxel resolution.

### MRI evaluation of oxidative stress

**Figure 1:**
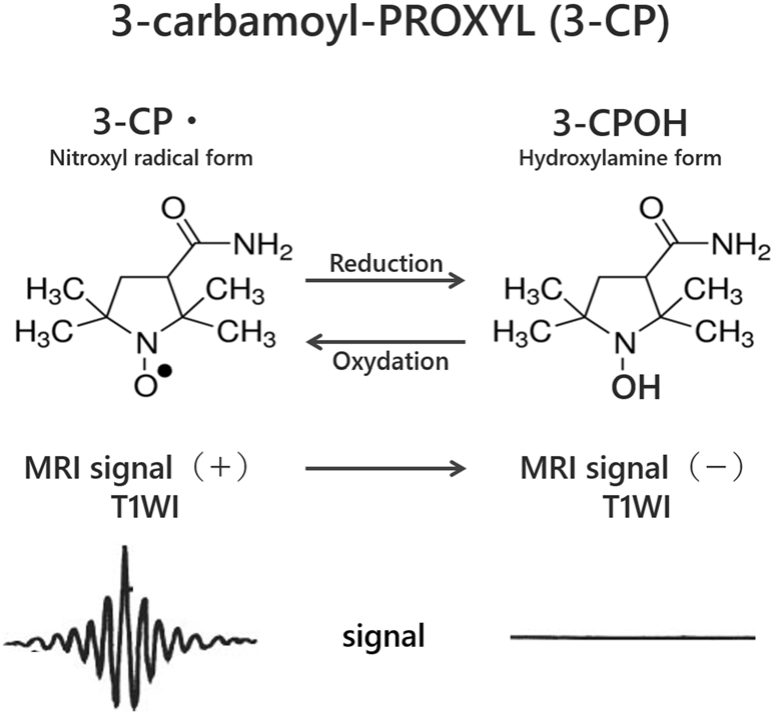
Structure and MRI signal of 3-carbamoyl-PROXYL (3-CP) and the corresponding hydroxylamine (3-CPOH). 3-CP is a nitroxyl radical that is chemically stable and has extremely low toxicity. It is paramagnetic and emits a high signal on T1WI MRI. 3-CP gets converted to a hydroxylamine form by reduction and the magnetic resonance imaging (MRI) signal is lost. The hydroxylamine form returns to 3-CP through oxidation and the MRI signal is produced again.

3-CP (3-carbamoyl-PROXYL; Sigma-Aldrich, St. Louis, MO, USA) is a nitroxyl radical having an unpaired electron and reveals high SI on the T1-weighted MRI images (T1WI) because of its paramagnetism (Fig. 1). 3-CP is reduced and becomes a hydroxylamine form and loses the MRI signal. The hydroxylamine form is oxidized by ROS and becomes 3-CP again. 3-CP was injected via a tail vein as a probe.

**Figure 2:**
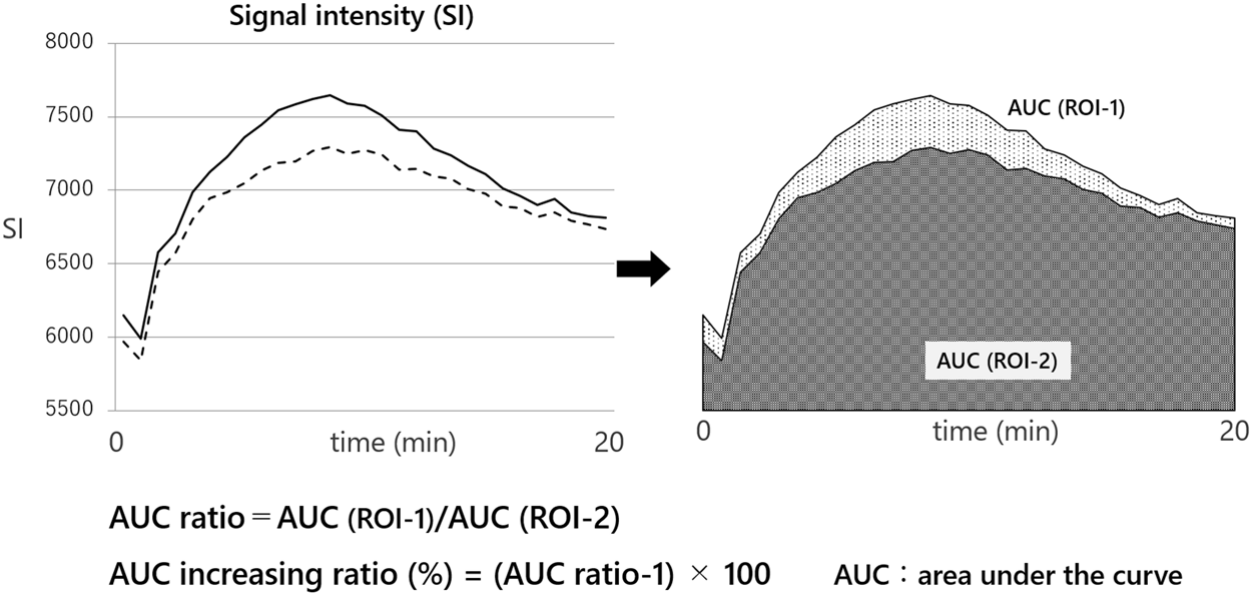
How to calculate the AUC ratio. The area under the curve (AUC) of signal intensity (SI) was calculated during MRI examination for 20 min. The AUC ratio and AUC increasing ratio were calculated using the following formulae: AUC ratio = AUC(ROI-1)/AUC(ROI-2) and AUC increasing ratio = (AUC ratio-1) × 100 (%).

MRI data were analyzed using Extended MR WorkSpace 2.6.3.5 (Philips, Amsterdam, Netherlands). The region of interest (ROI) was defined as the gastrocnemius muscles of the ischemia–reperfusion injury limb (ROI-1) and intact limb (ROI-2). The SI of the ROI-1 and ROI-2 were compared^9^. The SI graph at the ROI was created during MRI experiment and the signal strengths of ROI-1 and ROI-2 were compared by calculating the area under the curve (AUC)^10^ (Fig. 2). The AUC was determined with integration for the 20-min duration of the examination. When the SI for ischemia–reperfusion muscle was higher than the SI for intact muscle, it was suggested that oxidative stress had occurred in the ischemia–reperfusion muscle^6^.

### MRI examination

Rats were divided into three groups (n = 6 each group): a 4-h ischemia-0.5-h reperfusion group (I), a 4-h ischemia-2-h reperfusion group (II), 4-h ischemia-24-h reperfusion group (III). Following induction of anesthesia, the animal was placed in a supine position. A 24G cannula was inserted via the tail vein. Immediately after the defined reperfusion time, control images were acquired before probe administration. MRI scans started immediately after 3-CP (275 mg/kg; 1.48 mmol/kg) injection via the venous line and continued for 20 min. The T1WI data were analyzed and the oxidative stress was evaluated by comparing the AUC of ROI-1 and ROI-2.

### The effect of edaravone

Edaravone (Mitsubishi Tanabe Pharma Corporation, Osaka, Japan) is a radical scavenger and used as a treatment drug for cerebral infarction^11^. We examined whether edaravone could inhibit skeletal muscle injury after the ischemia–reperfusion experiment. A rat model subjected to 4 h of ischemia and 24 h of reperfusion (n = 4) was created and samples of blood and skeletal muscle (gastrocnemius in both cases) were taken. In this protocol edaravone (9.0 mg/kg) was administered into the peritoneal cavity at the beginning of the reperfusion and 12 h later according to the report by Yamamura et al.^12^, although peritoneal cavity administration is an off-label use of edaravone.

Additional MRI examinations with edaravone were performed using the same protocol mentioned above (n = 6 each group) to evaluate whether edaravone could inhibit the increase in 3-CP SI by eliminating ROS. In this experiment, we adopted a model of strong oxidative stress resulting from 4 h of ischemia and 24 h of reperfusion.

### Statistical analysis

All statistical analyses were performed using the R statistical package version 3.1.0 (R Core Team (2014). R: A language and environment for statistical computing. R Foundation for Statistical Computing, Vienna, Austria, https://www.R-project.org/). Data were expressed as the mean ± standard deviation (SD). Statistical differences were analyzed using the *t*-test and *p*-values < 0.05 were considered statistically significant. The *t*-test was applied for the comparison of the mean values of the two paired groups, and for the untreated group and the treated groups.

## Results

### Plasma CK level

**Figure 3:**
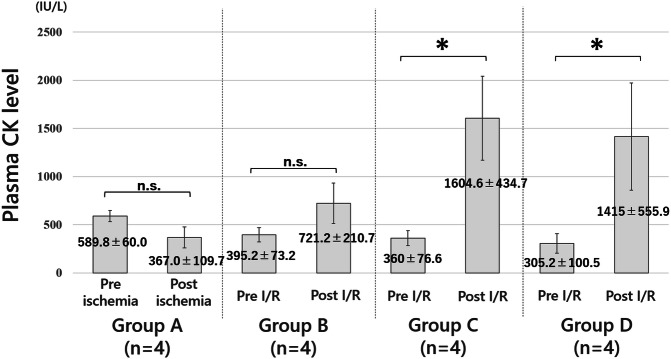
Plasma creatine kinase (CK) levels after ischemia/reperfusion. The plasma CK levels of group A (only ischemia), B (0.5-h reperfusion), C (2-h reperfusion), and D (24-h reperfusion) were 367.0 ± 109.7 IU/L, 721.2 ± 210.7 IU/L, 1604.6 ± 434.7 IU/L, and 1,415 ± 555.9 IU/L, respectively. *P* values versus preischemia–reperfusion are shown, and there was a significant difference between the intact limb and the ischemia–reperfusion limb in group C and D. *, *p*-value < 0.05; n.s., not significant.

Figure 3 shows the plasma CK levels of each group. The mean plasma CK level in all rats pre-ischemia was 411.2 ± 139.7 IU/L. Post-ischemia or ischemia–reperfusion plasma CK levels were 367.0 ± 109.7 IU/L [t(3) = -3.8429, *p* = 0.031] in group A, 721.2 ± 210.7 IU/L [t(3) = -2.8173, *p* = 0.067] in group B, 1604.6 ± 434.7 IU/L [t(3) = − 6.1754, *p* < 0.005] in group C, and 1,415 ± 555.9 IU/L [t(3) = − 4.3088, *p* < 0.05] in group D (Fig. 3). There were no drop-outs animals in groups A, B, C, or D.

## Histopathology

**Figure 4:**
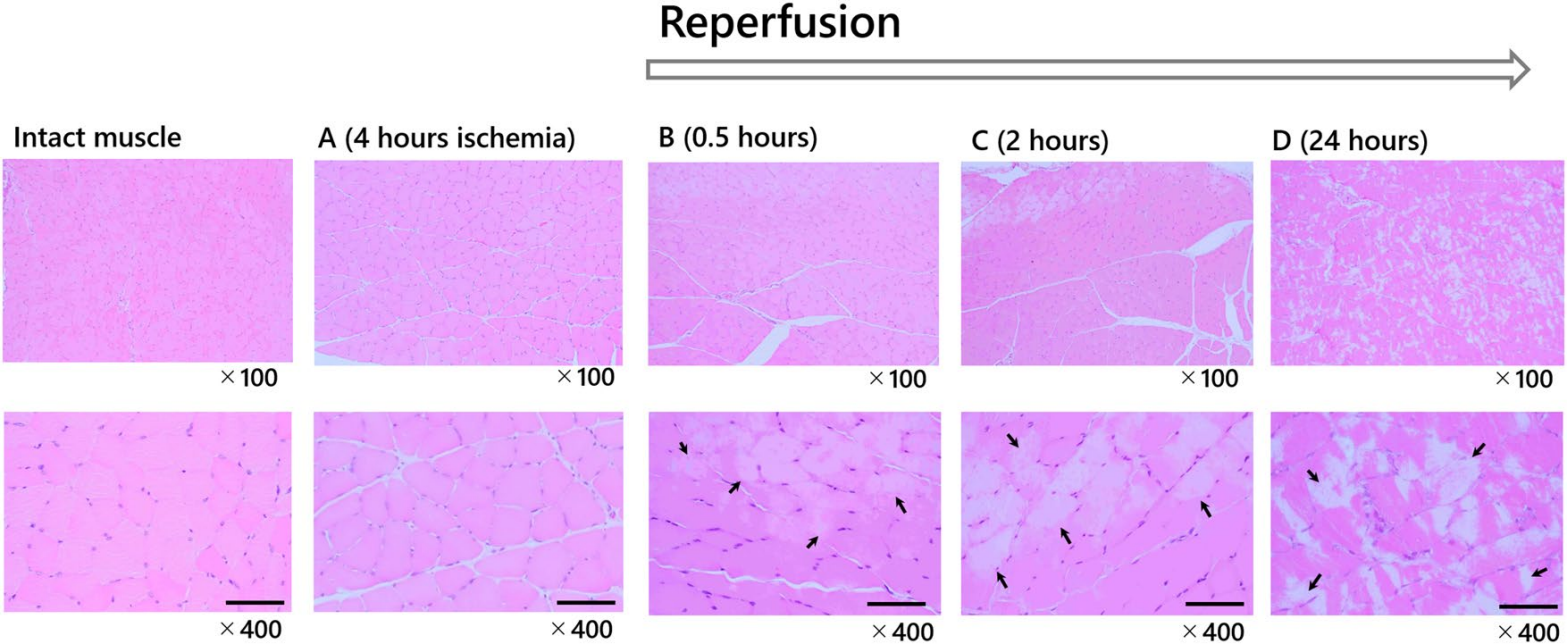
Histopathological findings (hematoxylin and eosin stain). Typical histopathological findings of each group were shown. The arrows indicate the injured area, and the bars represent 100 μm. Intact muscle group: no ischemia. Group A: only ischemia. Group B: 0.5-h reperfusion. Group C: 2-h reperfusion. Group D: 24-h reperfusion. There was no muscle destruction in both the intact muscle group and in group A. The longer the reperfusion time, the stronger the muscle destruction, including edema of cells, necrosis, and destruction of muscle structure occurred.

The degree of skeletal muscle injury was evaluated. Figure 4 demonstrates the typical histological images of each group as follows: intact muscle, group B, C, and D. There were no damaged cells observed in either the intact muscle group or group A. The extent of histological injury was 9.5 ± 1.6% in group B, 12.7 ± 3.7% in group C, and 43.6 ± 5.1% in group D. The duration of reperfusion was directly proportional to the destruction of muscle cells and muscle structure (Fig. 4).

### The evaluation of oxidative stress using MRI

**Figure 5:**
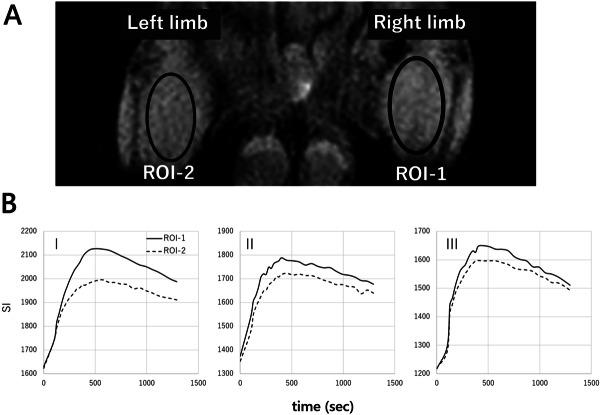
Time-course changes in signal intensity (SI) at region of interest (ROI). A representative MRI image used in the analysis (**A**). The image was obtained approximately 500 s after intravenous injection of 3-CP. The SI promptly reached a peaked and gradually decreased in all rats (**B**). The SI of ROI-1 was higher than that of ROI-2 in all groups after ischemia–reperfusion (**B**).

Figure 5A shows a representative MRI image used in the analysis. MRI image was obtained approximately 500 s after intravenous injection of 3-CP. The MRI SIs of ROI-1 and ROI-2 after intravenous injection of 3-CP were compared. We defined AUC ratio = AUC (ROI-1)/AUC (ROI-2) and AUC increasing ratio (%) = (AUC ratio-1) × 100 (Fig. 2). The AUC of SI was calculated.

**Figure 6:**
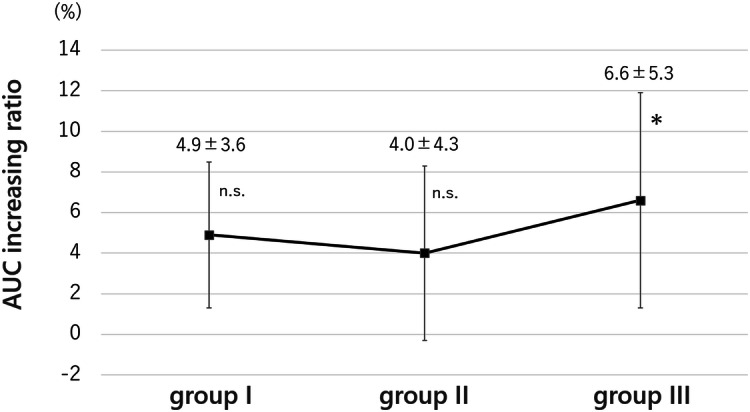
Area under the curve (AUC) increasing ratio. AUC increasing ratio was calculated using the formula; AUC increasing ratio = (AUC ratio-1) × 100 (%). AUC increasing ratio of group I, II, and III were 4.9% (*p* = 0.07), 4.0% (*p* = 0.11), and 6.6% (*p* < 0.05) respectively. The SI of ischemia–reperfusion limb was stronger than that of intact limb. *P* values versus intact limb are shown, and there is significant difference in group III. *, *p*-value < 0.05; n.s., not significant.

Figure 5B demonstrates the change in SI at the ROI-1 and ROI-2 of each group. The SI of ROI-1 was higher than that of ROI-2 in all groups (Fig. 5B). The AUC increasing ratio of group I, II, and III were 4.9 ± 3.6% [t(5) = 2.7687, *p* = 0.07], 4.0 ± 4.3% [t(5) = 2.0856, *p* = 0.11), and 6.6 ± 5.3% [t(5) = 2.7714, *p* < 0.05] respectively (Fig. 6). The SI of the ischemia–reperfusion limb was stronger than that of the intact limb in all three groups. There were no drop-outs animals in groups I, II, and III.

### The effect of edaravone for skeletal muscle injury

**Figure 7:**
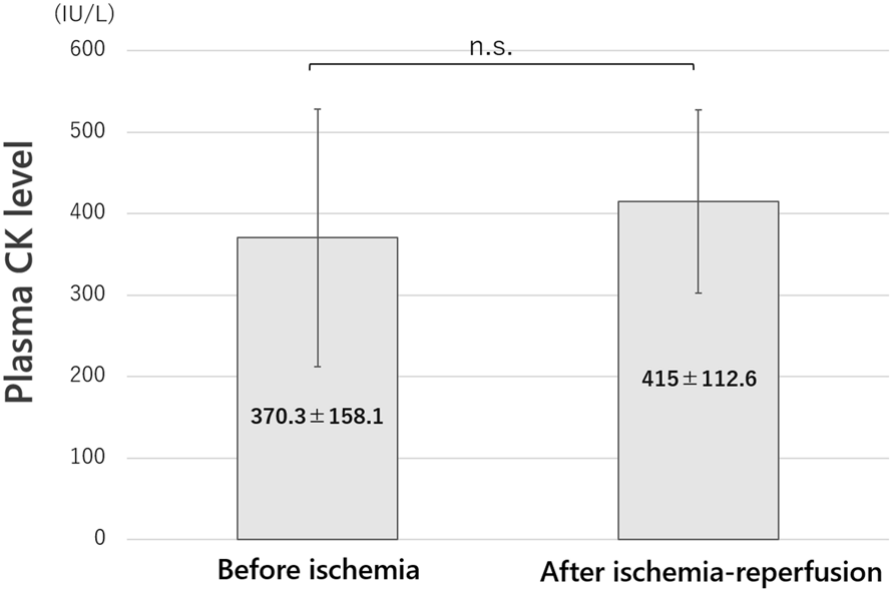
Plasma creatine kinase (CK) levels after ischemia–reperfusion with edaravone administration. Edaravone was administered to the 4-h ischemia-24-h reperfusion group. The pre-ischemia and post-reperfusion plasma CK levels were 370.3 ± 158.1 IU/L and 415 ± 112.6 IU/L, with no significant difference (*p* = 0.42). Note: Fig. 3 demonstrates the increase of the plasma CK level in group D (24-h reperfusion). n.s., not significant.

**Figure 8:**
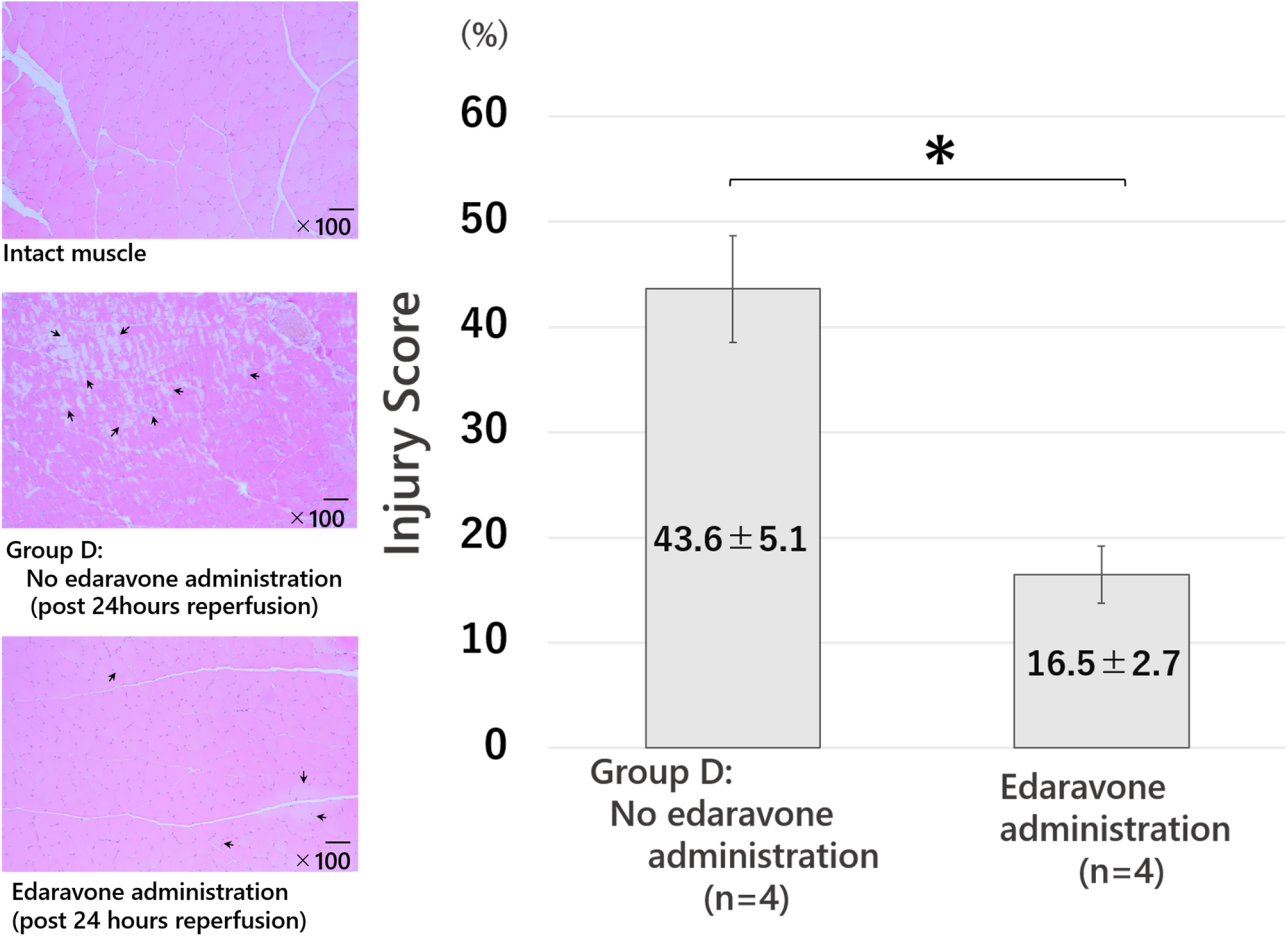
Histopathological findings in the edaravone group (hematoxylin and eosin stain). The arrows indicate the injured area, and the bars represent 100 μm. Edaravone administration group was compared with the group D as no administration group. The administration of edaravone reduced necrosis and swelling of skeletal muscle cells and reduced destruction of muscle structure. *, *p*-value < 0.05.

The plasma CK levels with edaravone administration before and after ischemia–reperfusion were 370.3 ± 158.1 IU/L and 415 ± 112.6 IU/L, respectively [t(3) = − 1.0033, *p* = 0.42] (Fig. 7). The plasma CK level in Group D (the same reperfusion time as the edaravone group) was 1,415 ± 555.9 IU/L and the elevation of CK level in the edaravone group was inhibited compared with that of group D. Furthermore, the extent of histological injury was 43.6 ± 5.1% in group D and 16.5 ± 2.7% in the edaravone group, and the histopathological evaluation showed that edaravone significantly suppressed the degree of myocyte injury (*p* < 0.05, Fig. 8). There were no drop-outs animals in edaravone-administrated and non-administrated groups.

### The effect of edaravone on oxidative stress

**Figure 9:**
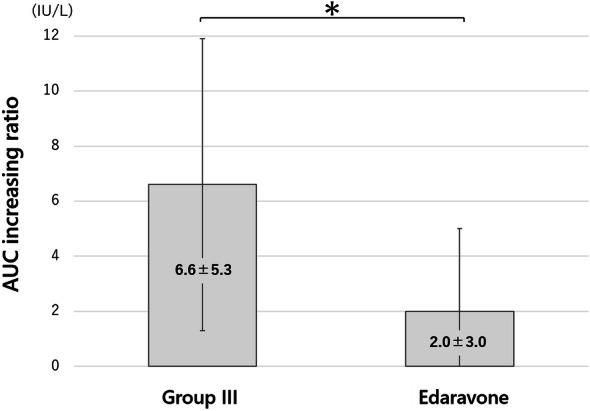
Area under the curve (AUC) ratio in the edaravone group. AUC ratio was thought to express oxidative stress. Edaravone group was compared with the group III as no administration group. The AUC increasing ratio in the edaravone group was lower than that of group III (*p* < 0.05). *, *p*-value < 0.05.

Twenty-four after reperfusion, edaravone administration reduced the AUC (ROI-1) for the ischemia–reperfusion limb, which was not significantly different from the AUC (ROI-2) for the intact limb (*p* = 0.21). Twenty-four hours after reperfusion, the ratio of increase in the AUC was 6.6 ± 5.3% in the non-administration group and 2.0 ± 3.0% in the edaravone administration group, the difference being significant (*p* < 0.05). These results suggest that edaravone inhibited tha increase in the AUC reflecting oxidative stress, in ischemia-reperfused hindlimbs (Fig. 9). In the experiment, there were no drop-out animals in either of the groups.

## Discussion

We investigated the feasibility of the detection of oxidative stress in the skeletal muscle in an ischemia–reperfusion injury model using 3-CP enhanced MRI. According to the ARRIVE guidelines, we randomly grouped the rats and assessed the data with mean ± SD to eliminate biases as much as possible. We also evaluated plasma CK levels and histopathology and examined the relationship between the degree of organ injury and MRI findings. T1WI MRI can detect the 3-CP signal in real time, and oxidative stress can be evaluated based on the increase in the SI of the 3-CP.

CK is an enzyme mainly distributed in the skeletal muscle cells and cardiomyocytes, and it is known to leak into the blood with muscle cell injury. The plasma CK level therefore elevates with ischemia–reperfusion injury of skeletal muscle^13^. However, this increase is not immediate. It sometimes takes a few days to reach peak plasma CK level after ischemia–reperfusion injury. In the present study, as group A demonstrated, the plasma CK levels did not elevate in the no-reperfusion group; however, in the other reperfusion groups, the plasma CK levels elevated and tended to be higher as the reperfusion time increased. The histopathological findings of ischemia–reperfusion injury of skeletal muscle vary. Necrosis or apoptosis occur after strong ischemia–reperfusion, but weak ischemia–reperfusion induces only edema of cells and intercellular stroma^12^. In our rat model, destruction of skeletal muscle was induced, and the degree of injury became stronger in direct proportion to the reperfusion time. In the edaravone group, the plasma CK levels were not elevated and the destruction of skeletal muscle was weak on histopathological analysis. As to the protective effect of edaravone, it will be necessary to further examine not only the degree of skeletal muscle injury, but also motor ability of the hind limb after ischemia–reperfusion. Edaravone is widely used for treatment of the acute phase of cerebral infarction. Histological findings indicated that edaravone significantly inhibited the increased plasma CK levels and muscle injury after limb ischemia–reperfusion, suggesting that it may be clinically applicable for treatment of acute arterial occlusion of the extremities.

Ischemia–reperfusion injury of skeletal muscle may result in limb dysfunction, amputation, and even death^14^. Although various factors are involved, ROS are believed to play a central role in ischemia–reperfusion injury. ROS are capable of reacting with and damaging various molecular targets including DNA, proteins, and lipids. More importantly, ROS have been found to act as signaling molecules in various cellular signaling pathways^15^. Under various pathological conditions such as ischemia–reperfusion, excess amounts of accumulated ROS induce apoptosis or necrosis by activating the mitogen-activated protein kinase, caspase cascades, and/or by disrupting mitochondrial function^16^. This contribution of ROS to apoptosis and necrosis is highly cell-type specific and depends on the amount of endogenously or exogenously generated ROS present. In the present study, we showed that ROS were involved in skeletal muscle injury after ischemia–reperfusion. Further investigation is necessary to clarify the roles of apoptosis and necrosis in skeletal muscle cell death caused by ROS.

ROS are produced by ischemia–reperfusion of skeletal muscle. Ischemia–reperfusion injury consists of two phases: early-phase injury due to xanthine oxidase or mitochondria-derived ROS (within several hours after reperfusion) and late-phase injury due to secondary production of ROS by infiltrating inflammatory cells (peaking at 24 h after reperfusion). Group (B) and (C) correspond to early-phase muscle injury, and group (D) to late-phase muscle injury^17^.

MRI examination with 3-CP was performed continuously for 20 min. The ischemia–reperfusion limb and intact limb of the same rat were compared on T1WI. Although the SIs of the ischemia–reperfusion limb tended to be higher than that of the intact limb in all groups, there was only a significant difference in group III (24-h reperfusion group). Furthermore, in the edaravone group, the elevation of SI tended to be smaller than in the other groups. Early detection of oxidative stress associated with various diseases is very useful. It can be applied in a wide variety of clinical settings and has the potential to positively influence the treatment of numerous diseases.

The redox state, i.e., production of free radicals including ROS and the activity of the antioxidant system, is well balanced in organs and tissues under physiological conditions. However, redox balance collapses during various organ injuries such as cancer and ischemia. That is, the oxidative stress implies overproduction of ROS^1,3^. It is technically impossible to diagnose oxidative stress using only visual modalities. Some basic studies have attempted detection of radicals using ESR^18,19^. However, radical detection has proven to be problematic because radicals are extremely chemically unstable and their elimination time is short. 3-CP is one redox-sensitive probe that has been used for an ESR study, as it is chemically stable radical and with extremely low toxicity^20^.

MRI is a common clinical imaging modality. Moreover, paramagnetic 3-CP has the characteristic spin probe of MRI and emits high SI in T1WI^5,21–23^. Zhelev et al. reported that signal elevation after injection of 2,2,6,6-tetramethylpiperidine 1-oxyl, a nitroxyl radical, indicates oxidative stress^6^. When the SI of 3-CP in the ischemia–reperfusion limb is higher than that of the intact limb, it suggests that oxidative stress has occurred in ischemia–reperfusion limb. In our model of limb ischemia–reperfusion, we were able to assess oxidative stress status in vivo in real time. MRI is superior for spatiotemporal analysis and can image toxic molecules, thus making it an effective tool for devising and evaluating treatments for ischemia in parenchymal organs. Our study had two limitations. First, due to the small number of rats used in the experiments possible biases must be considered even without animal dropouts. Second, blood flow after ischemia–reperfusion was not measured. It has been reported that 80% blood flow is resumed soon after reperfusion without heparinization^24^. Although we should be cautious about drawing any conclusions, we have established an in vivo method for evaluating oxidative stress, i.e. organ damage-related molecules in diseased area, and therefore our study may represent a cornerstone for clinical application.

In the edaravone group, the AUC increasing ratio was small and there was no significant difference between the ischemia–reperfusion limb and intact limb. Moreover, the plasma CK level elevation was inhibited and destruction of muscle cells was reduced. In our study, it was suggested that edaravone worked as a radical scavenger at the skeletal muscle level, inhibited oxidative stress, and reduced ischemia–reperfusion injury. Furthermore, edaravone as a radical scavenger reduced elevation of the AUC increasing ratio; this result provided evidence that 3-CP enhanced MRI could evaluate the oxidative stress at a local injury site.

## Conclusion

To the best of our knowledge, ischemia–reperfusion injury has not been diagnosed using an imaging modality. The treatment strategy is decided based on the symptoms and clinical course, which could cause potentially fatal delays in treatment. 3-CP enhanced MRI makes it possible to recognize oxidative stress in the skeletal muscle and provides the benefit of rapidly determining the ideal treatment strategy for ischemia–reperfusion injury.
